# Public Knowledge, Attitudes, and Practice towards COVID-19 Pandemic in Saudi Arabia: A Web-Based Cross-Sectional Survey

**DOI:** 10.3390/medsci9010011

**Published:** 2021-02-16

**Authors:** Ali Hassan A. Alnasser, Jaffar A. Al-Tawfiq, Mohammed Sheker H. Al-Kalif, Rubayyi Faris B. Shahadah, Khawlah Saad A. Almuqati, Bashayer Sulaiman A. Al-Sulaiman, Khulud Khalid S. Alharbi, Fatimah Yousef M. Alabbad, Jamilah Yousf M. Alabbad, Ibrahim Abdulrahman I. Alquwaiz, Ibrahim Khalil I. Almashama

**Affiliations:** 1 Department of Laboratory, Dhahran Eye Specialist Hospital, Ministry of Health, Dhahran 31942, Saudi Arabia; 2 Infectious Disease Unit, Specialty Internal Medicine, Johns Hopkins Aramco Healthcare, Dhahran 31311, Saudi Arabia; jaltawfi@yahoo.com; 3 Division of Infectious Diseases, Department of Medicine, Indiana University School of Medicine, Indianapolis, IN 46202, USA; 4 Division of Infectious Diseases, Department of Medicine, School of Medicine, Johns Hopkins University, Baltimore, MD 21287, USA; 5 College of Public Health, Imam Abdulrahman Bin Faisal University, Dammam 31441, Saudi Arabia; moudshk@gmail.com; 6 Technical and Vocational Training Corporation, Riyadh 11472, Saudi Arabia; 7 College of Dentistry, Taibah University, Madinah 42313, Saudi Arabia; rabeafs20@gmail.com; 8 King Faisal Specialist Hospital and Research Centre (Gen. Org.), Riyadh 11211, Saudi Arabia; khawlah.almuqati@gmail.com; 9 First Health Cluster in Eastern Province, Dammam 31444, Saudi Arabia; bashayer.sulaiman@hotmail.com; 10 College of Applied Medical Science, Umm Al-Qura University, Makkah 21955, Saudi Arabia; khuludk.alharbi@gmail.com; 11 The Panuska College of Professional Studies, The University of Scranton, Scranton, PA 18510, USA; 12 Department of Orthopedic Surgery, Dammam Medical Complex, Dammam 31463, Saudi Arabia; alabbad82@yahoo.com; 13 Department of Pediatrics, Anak General Hospital, Qatif 31911, Saudi Arabia; alabbadjamelah@gmail.com; 14 College of Medicine, Prince Sattam Bin Abdulaziz University, Al‐Kharj 11942, Saudi Arabia; 448.ibrahem@gmail.com; 15 College of Clinical Pharmacy, Imam Abdulrahman Bin Faisal University, Dammam 31441, Saudi Arabia; ikiaim@hotmail.com

**Keywords:** the coronavirus disease 2019, SARS-CoV-2, 2019-nCoV, awareness, public awareness, public practice, social distancing, preventive measures, perception, COVID-19, social media, KAP

## Abstract

(1) Background: COVID-19 has become a worldwide public health problem. No previous study has investigated factors associated with COVID-19 knowledge, attitude, and practice (KAP) after completely lifting the curfew in all Saudi Arabia regions and cities. Therefore, adequate knowledge, a positive attitude, and correct control of COVID-19 are essential to eradicate the disease. Hence, this study aims to assess factors associated with KAP of COVID-19; (2) Methods: This cross-sectional web-based survey was performed with the participation of 4305 individuals aged over 15 years living in Saudi Arabia from 11 to 19 August 2020. They were included using the snowball sampling method; (3) Results: Of the 4305 participants, 94.9% were Saudis, 60% females, and 45.4% were in the age group of 20–34 years, 61.7% married, and 49.3% from the Eastern Province of Saudi Arabia. Most of the participants demonstrated good KAP levels (89.6%, 87.2%, and 87.2%) towards the COVID-19 pandemic, respectively. In addition, most of the participants (85.8%) used the internet and social media as a source for COVID-19 information (4) Conclusions: The finding showed that most of the participants demonstrated good knowledge of COVID-19, positive attitudes, and demonstrated good practices for preventing the spread of disease infection.

## 1. Introduction

Coronaviruses (CoVs) are respiratory viruses and belong to a large family of RNA viruses known for more than 55 years [1]. Moreover, these viruses affect a wide variety of hosts and result in a wide range of disease severity from the common cold to more deadly disease [1]. Recently, severe acute respiratory syndrome (SARS) and the Middle East respiratory syndrome (MERS) emerged and caused high mortality [2]. At the end of December 2019, in Wuhan city, China, a cluster of acute respiratory disease cases caused by an unknown pathogen was reported [2,3]. Subsequently, the virus was identified as a novel coronavirus on 7 January 2020 [3] and was designated as severe acute respiratory syndrome coronavirus 2 (SARS-CoV-2), and the disease was called Coronavirus disease 2019 (COVID-19) [3]. SARS-CoV-2 causes a wide range of symptoms from dry cough, fever, shortness of breath, and fatigue [4,5,6]. The SARS-CoV-2 virus remains on surfaces for a few hours to several days, depending on the environmental conditions [7].

SARS-CoV-2 has caused outbreaks in hospitals and led to increased morbidity and mortality rates [1]. Globally, on 13 February 2021, there were a total of 107,686,655 confirmed cases of COVID-19, including 2,368,571 deaths [8].

The Kingdom of Saudi Arabia is the largest country in the Arabian Gulf region and has a population of more than 34 million. Initial COVID-19 cases in this area were travel-related cases from travelers from Iran and Iraq to Bahrain and Kuwait [9,10]. Similarly, the first COVID-19 case in Saudi Arabia was reported in Qatif, Eastern Province, in a returning traveler [9,11]; that case was reported on 2 March 2020 [3]. Saudi Arabia took multiple interventions to prevent the spread and transmission of SARS-CoV-2, such as canceled mass gatherings, schools’ closure, and limiting Hajj pilgrimage, curfew, and lockdown of localities with increased transmission [9,10,11]. As of 13 February 2021, in Saudi Arabia, there were 372,073 confirmed cases of COVID-19 with 6,424 deaths [8].

Misconceptions about infection prevention and treatment persist among the public. They are limited studies exploring knowledge, attitudes, and practices (KAP) among the general population during the COVID-19 pandemic in the Middle East and Saudi Arabia. To our knowledge, there are two studies [2,7] from Saudi Arabia on KAP among citizens and residents, conducted during curfews and city closures. In one of these studies, most of the included population had high knowledge, optimistic attitudes, and good practices [2]. Another study showed KAP of COVID-19 was related to educational level [7]. An additional study showed a similar finding, and there were multiple myths as well [12]. A recent study showed that 93.7% of the surveyed population in Saudi Arabia were aware of asymptomatic carriers and that the majority were aware of preventive measures [13]. Other studies about KAP in relation to COVID-19 in Saudi Arabia were related to healthcare workers and not the general population. In addition, there is no previous research investigating factors associated with COVID-19 KAP after lifting the country-wide curfew of the Kingdom of Saudi Arabia. Therefore, we conducted this study of KAP after lifting these measures and compared the results with previous studies of the COVID-19 KAP.

## 2. Design and Methods

### 2.1. Study Area, Design, and Period

This web-based cross-sectional survey was conducted over nine days from 11 August to 19 August 2020, several weeks after the complete lifting of curfew in all regions of Saudi Arabia. 

### 2.2. Study Participants

This snowball sampling study targeted adult citizens and Saudi Arabia residents aged 15 years or over who were in Saudi Arabia during the COVID-19 pandemic.

### 2.3. Questionnaire Design

The questionnaire was planned to evaluate the KAP of the Saudi MOH guidelines for awareness and prevention of the COVID-19 pandemic. The questionnaire was intended to identify opportunities for improvements in COVID-19 awareness, assess the practices that potentially contribute to the persistence of COVID-19 in Saudi Arabia, and assess the community’s attitudes towards the COVID-19 pandemic. The study’s authors team developed a questionnaire of 20 closed-ended type questions with multiple choice answers. The questionnaire was available in both English and Arabic languages.

The questionnaire has five parts. The first part was about the participant’s sociodemographic data and has seven items (Nationality, Gender, Age, Marital status, Place of living, Educational level, Occupational status). The second part has four items and assessed knowledge about the COVID-19 pandemic. Each item had multiple choices with correct choices and other incorrect choices. These questions included the most affected groups, typical symptoms of COVID-19, the most common modes of transmission of the SARS-CoV-2 virus, and the needed self-protection measures to reduce the possibility of COVID-19 infection. The third part has four items about the attitude towards the COVID-19 pandemic. Attitudes’ items have a 5-point Likert scale with a positive direction assessing the attitude of participants to the following: reducing the number of pilgrims during the Hajj season will reduce the number of COVID-19 cases, the need to wear face masks to protect themselves from COVID-19 infection, COVID-19 will be successfully controlled, and individuals are committed to the precautions set by the Ministry of Health. The fourth part assessed the practice of the participants to prevents SARS-CoV-2 transmission. Each part has four questions and a 5-point Likert scale (1 strongly disagree to 5 strongly agree) and included avoiding going to crowded places, wearing a face mask when going outside, hand washing with soap and water, and abiding with no handshaking. The last part probed the source of information the participant uses to get updates about the COVID-19 pandemic.

The questionnaire was pre-tested, and 85 people from all Saudi Arabia regions responded from 30 July to 2 August 2020 to test the questionnaire’s validity and reliability and ensure clarity and understanding of all elements. The relevant opinions of the participant were considered, and adjustments were applied. Cronbach coefficient α was used to test the questionnaire’s internal consistency was higher than (0.7). Responses from the pilot study were not included in the final study results.

### 2.4. Data Collection

A snowball sampling method was used, and data were collected through a self-questionnaire using (Google forms). The questionnaire's link was distributed via social media like WhatsApp, Twitter, Facebook, and Instagram. The response was received from all Saudi Arabia regions.

### 2.5. Statistical Analysis

The study data were collected via (Google Forms) and then transferred to an (Excel sheet). Then, data were cleaned, coded, and measured for reliability and validity. The Statistical Package for the Social Sciences (SPSS version 25.0; IBM Corp., Armonk, NY: USA) was used to describe the basic features of the data in the study through frequencies, percentages. 'Spearman's Rho is a nonparametric measure of rank correlation, denoted by the Greek letter ‘ρ’ (Rho) measures the strength and direction of the association between two ranked variables, which appropriate for both continuous and discrete ordinal variables. Spearman's rank coefficient was used for the association between knowledge, attitude, and practices. The strength of the association is described as follows: 0.0–0.19 “very weak,” 0.20–0.39 “weak,” 0.40–0.59 “moderate,” 0.60–0.79 “strong,” and 0.80–1.0 “very strong”. For all tests, a *p*-value < 0.05 was taken as statistically significant. KAP’s total scores were categorized into “good” and “poor” scores based on an 80% cut-off point out of the total.

## 3. Result

### 3.1. Sample Characteristics

A total of 4305 individuals responded to the online survey. Most of the study respondents were Saudi (94.9%, N = 4084). More than half of the study respondents (60%, N = 2583) were female, and 45.4% (N = 1954) were in the age range of 20–34; more than half of the study respondents 61.75% (N = 2658) were married, and around half of the study respondents 49.3% (N = 2123) were residents from the Eastern Province. Of the respondents, one-third of the study respondents, 33.7% (N = 1452), were residents from the Western Province of Saudi Arabia. About education, 57.2% (N = 2461) had bachelor’s degrees, and 29.2% (N = 1255) had high school/diploma. In relation to the job of respondents, 32.9% (N = 1416) were non-healthcare practitioners, and 30.6% (N = 1317) were unemployed (Table 1).

### 3.2. Awareness and Knowledge of COVID-19

**Figure 1:**
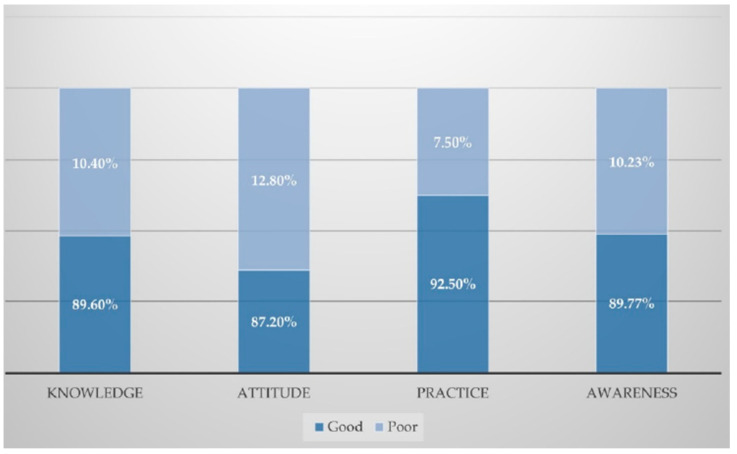
Awareness and overall KAP levels regarding the COVID-19 pandemic in Saudi Arabia.

**Figure 2:**
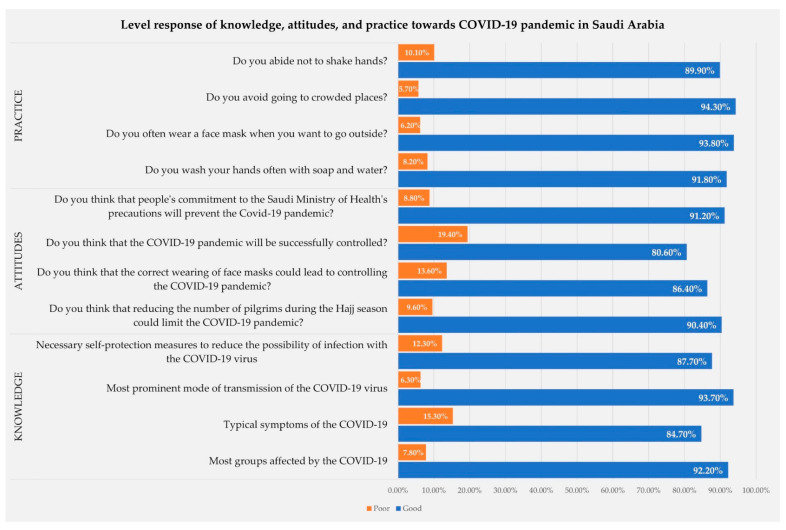
The level response of knowledge, attitudes, and practice towards COVID-19 pandemic in Saudi 1 .

Figure 1 shows that most of the responders 89.7% had a good level of awareness towards the COVID-19 pandemic. Besides, 89.6% of responders had good knowledge about the COVID-19 pandemic, and 93.7% of responders had good knowledge about the most prominent modes of transmission of COVID-19. Good knowledge was observed in 92.2% about the most affected groups, 87.7% about self-protection measures, and 84.7% about typical symptoms (Figure 1 and Figure 2).

### 3.3. Association between Knowledge and Demographic Variables

Table 1 shows the association between knowledge about the COVID-19 pandemic and demographic variables. There is a statistically significant association between knowledge about the COVID-19 pandemic and place of living (*p* < 0.05) in favor of those participants from the Western Province of Saudi Arabia.

### 3.4. Attitude towards the COVID-19 Pandemic

Figure 1 shows overall good attitudes towards the COVID-19 pandemic were seen in 87.2% of the participants. Besides, 91.2% of participants agreed that an individual’s commitment to the precautions of the Saudi Ministry of Health (MOH) will prevent the spread of COVID-19. However, 90.4% of participants think that reducing the number of pilgrims during the Hajj season could limit the pandemic of COVID-19. Furthermore, 86.4% believe that the correct wearing of face masks could lead to controlling the pandemic. Additionally, 80.6% of participants think that COVID-19 will be successfully controlled (Figure 1 and Figure 2).

### 3.5. Association between Attitude and Demographic Variables

Table 1 shows the association between attitude towards COVID-19 pandemic and demographic variables. There is a statistically significant association between participants’ attitudes regarding the COVID-19 pandemic in favor of married participants, Western Province, and bachelor’s degree.

### 3.6. Practice towards the COVID-19 Pandemic

Figure 1 shows that 92.5% of participants had good practices regarding the COVID-19 pandemic. Of the participants, 94.3% avoid going to crowded places, 93.8% often wear a face mask when outside, 91.8% wash their hands often with soap and water, and 89.9% abide not to shake hands (Figure 1 and Figure 2).

### 3.7. Association between Practices and Demographic Variables

Table 1 shows the association between practice about the COVID-19 pandemic and demographic variables. There is a statistically significant association between attitude towards COVID-19 pandemic and nationality, gender, age, marital status, place of living, educational level, and occupational status (*p* < 0.05) in favor of (Saudi, female, 35–50 years, married, Eastern Province, bachelor’s degree, and employee non-a-health-practitioner) that had good practices.

### 3.8. Correlation between Knowledge, Attitude, and Practice

Spearman’s Rho tests results showed a statistically very strong significant association between knowledge, attitude, and practices (*p* < 0.01). The highest correlation coefficient was found between attitude and practice by 0.988, followed by 0.987 for the relation between practice and knowledge, followed by 0.976 for the relationship between knowledge and attitude.

### 3.9. Information Sources about COVID-19 Pandemic

The sources of respondents’ information about the COVID-19 pandemic were the internet/social media (85.8%), health practitioners (54.7%), TV/Radio (35.7%), family/friends (29.5%), and other sources (7%).

## 4. Discussion

This study explored public awareness among citizens and residents of the Kingdom of Saudi Arabia. Also, the study assessed knowledge, attitudes, and practices towards the COVID-19 pandemic. This study was conducted several weeks after the complete lifting of the curfew in all regions and cities of the Kingdom of Saudi Arabia. Besides, all economic and commercial activities return. However, there were additional protocols to adhere to social distancing, wearing a mask, and avoiding gatherings of more than 50 people.

This study included 4305 participants, most of whom were well-versed in knowledge related to COVID-19, and demonstrated a positive attitude, and proactive practices during the COVID-19 pandemic. The finding is consistent with previous studies associated with the COVID-19 pandemic [2,7,12,13]. This study also indicates that Saudi governmental health websites are a useful source of information for the public. The primary source of information about the COVID-19 pandemic was the internet and social media. This finding is similar to a study conducted in Egypt, and showed that the most common media were social media, and TV (80.8% and 35.1%, respectively) [5]. However, social media dependence varies in relation to different countries, and may be dictated by its norms and social norms. In a study from North-Central Nigeria study, participants’ knowledge was gained mainly through the internet/social media (55.7%) and Television (27.5%) [6]. Another study from India showed that 66.3% of participants acquired information from social media, and 17.4% from TV [14]. In another Ethiopian study, 59.9% of participants acquired information from TV/Radio [15].

Additionally, our findings showed that most study participants had a good awareness level (89.77%) regarding the COVID-19 pandemic. Furthermore, the study participants achieved a high level of knowledge with a mean score of 89.6% among participants, particularly from the Western Province of Saudi Arabia. This good knowledge could be attributed to the intensive health awareness promotions as launched by the Saudi MOH. Also, the observation of good knowledge could be explained by the fact that 66.2% of the participants had a bachelor’s degree.

The current study showed a good level of knowledge (89.6%), a good attitude (87.2%), and practice (92.5%), compared to a previous study [6]. Moreover, most (93.7%) of the study participants were aware of the most prominent modes of transmission of SARS-CoV-2. This finding is supported by other studies showing that 95% had good knowledge [16,17,18]. On the other hand, Ethiopian research showed poor knowledge about disease transmission (42.4%) [19].

Our findings revealed that 92.2% of the participants were unaware of the age groups most affected by COVID-19, similar to another study from India [20], but differs from an Egyptian survey that showed older people had good knowledge about the main at-risk category [5]. Good knowledge about the needed self-protection measures to reduce the possibility of infection with SARS-CoV-2 was 87.7% in the current participants. This finding is different from the 73% rate in a study from Pakistan [21]. This variation in the knowledge is related to other countries' sociodemographic and is probably reflected in the knowledge about typical COVID-9 symptoms. In the current study, 84.7% of the participants had good knowledge about typical symptoms of COVID-19, like other studies [2,7,14,20,22,23]. On the other hand, other studies showed <80% had poor knowledge about typical symptoms of COVID-19 [16,17].

Concerning to study responders’ attitude towards the COVID-19 pandemic in Saudi Arabia, the highest level of good attitude about the COVID-19 pandemic was among married participants and those from the Western Province of Saudi Arabia with bachelor’s degrees. This finding is similar to other studies with higher KAP among married individuals [24,25]. It was thought that married individuals had a higher level of positive attitudes towards COVID-19 as they cared for close family members, including young children [24].

Of the participants, 91.2% thought that people’s commitment to the Ministry of Health’s precautions will prevent the COVID-19 pandemic, as noted previously [26]. Of this study’s participants, 90.4% believed that reducing the number of pilgrims during the Hajj season could limit the COVID-19 pandemic in line with the recommendations [27], and 86.4% of participants believed that the correct wearing of face masks could lead to controlling the COVID-19 pandemic, similar to a study from Pakistan [28]. In contrast to the rate of 36.4% in a study from Egypt [29], 80.6% of study participants believed that COVID-19 will be successfully controlled, which is similar to studies from China, Tanzania, and Malaysia [23,30,31], and is different from a study from Pakistan that showed 77% believed that COVID-19 would be controlled successfully [28], and 53.5% of respondents in a study from India were not sure about the government response [14].

Concerning study responders’ practice towards COVID-19 pandemic in Saudi Arabia, the highest level of good practices regarding COVID-19 pandemic was among Saudis, females, and those with 35–50 years, being in Eastern Province, and those with bachelor’s degree. The study participants showed that 94.3% avoid going to crowded places, which is similar to a study conducted in India and China [14,23], and different from a study in Ethiopia, the Philippines, and the UK that showed 38.1%, 56.5%, and 62.9% of respondents avoided crowded places, respectively [15,32,33].

In this study, 93.8% of participants often wear face masks when they go outside, similar to what was reported from India, Vietnam, Tanzania and Ecuador [14,25,30,34]. However, this contrasts to other studies that reported less than 60% wearing a face mask when going out in public in other studies [15,16,31,32,35,36,37,38]. Hand hygiene was reported by 91.85% of study participants, similar to other studies showing (92–96.6%) of participants frequently practiced hand hygiene [14,16,34,39]. However, other studies showed <90% compliance with hand hygiene [15,28,32]. Furthermore, 89.9% of study participants did not shake hands. This finding is similar to previous studies from India and Egypt where 88.4% and 87% did not shake hands, respectively [14,16]. In contrast, 71.7% of Ethiopian participants in a previous study did not shake hands [15]. Hand hygiene could be a cultural and social practice in certain societies. Besides, it could be that people are hesitant to report low levels of handwashing, and this varies by cultural context for various reasons. It was shown that hand hygiene might be viewed as socially acceptable and mandated behavior rather than the basis of science [40]. Also, hand hygiene might be influenced by religious practices as it is in Saudi Arabia [41]. 

When interpreting this study’s results, it is essential to keep in mind that the current research has a large sample of recruited participants during this COVID-19 pandemic. However, some limitations should be considered. Most of the respondents were educated; therefore, the responses may not be generalizable to all the populations, including uneducated people. As in this study, self-reporting of data may also result in reporting bias, as in hand hygiene. Moreover, the questionnaire’s response is probably dependent on the authors’ networks, and this may have omitted useful comments from other people whom the questionnaire did not reach. This is substantiated by the fact that most of the respondents were from the Eastern and Western Provinces of Saudi Arabia, where most of the authors come from.

## 5. Conclusions

Our findings reveal that most of the respondents had a good knowledge of the COVID-19 pandemic, particularly those from the Western Province of Saudi Arabia. The study demonstrated a few unexpected negative attitudes and practices towards using protective measures. In particular, a few of the participants had different practices of shaking hands and not washing hands. Additionally, a small number of participants considered that COVID-19 will not be successfully controlled, and they thought that wearing masks is not effective in controlling the COVID-19 pandemic. The biggest sources of respondents’ information about the COVID-19 pandemic were the internet and health practitioners. Thus, providing effective health education programs may improve knowledge of COVID-19 and reduce negative attitudes and practices.

## Figures and Tables

**Table 1:** Association between knowledge, attitudes, and practices (KAP) and sociodemographic characteristics of the COVID-19 pandemic.

Characteristics		Total		Knowledge				Attitude				Practice			
Variables		N	%	Poor	Good	χ^2^	*p*-Value	Poor	Good	χ^2^	*p*-Value	Poor	Good	χ^2^	*p*-Value
Nationality	Saudi	4084	94.9	37.8%	62.2%	0.006	0.41	8.5%	91.5%	0.029	0.865	5.5%	94.5%	9.393	0.004 **
	Non-Saudi	221	5.1	37.6%	62.4%			8.1%	91.9%			5.5%	94.5%		
Gender	Female	2583	60.0	37.6%	62.4%	0.111	0.739	8.6%	91.4%	0.162	0.687	5.0%	95.0%	7.302	0.007 **
	Male	1722	40.0	38.1%	61.9%			8.2%	91.8%			6.9%	93.1%		
Age	15–19	291	6.8	41.6%	58.4%	4.925	0.177	10.0%	90.0%	2.492	0.477	11.3%	88.7%		
	20–34	1954	45.4	37.3%	62.7%			8.9%	91.1%			7.7%	92.3%		
	35–50	1485	34.5	36.6%	63.4%			7.7%	92.3%			3.0%	97.0%	58.551	0.000 **
	>50	575	13.4	36.6%	63.4%			8.0%	92.0%			3.1%	96.9%		
Marital status	Single	1475	34.3	39.1%	60.9%	1.809	0.613	8.7%	91.3%			8.7%	91.3%		
	Married	2658	61.7	37.1%	62.9%			8.1%	91.9%	9.487	0.023 *	4.3%	95.7%	37.067	0.000 **
	Divorced	129	3.0	36.4%	63.6%			9.3%	90.7%			2.3%	97.7%		
	Widowed	43	1.0	39.5%	60.5%			20.9%	79.1%			2.3%	97.7%		
Place of living	Central Province	597	13.9	35.0%	65.0%			8.5%	91.5%			9.4%	90.6%		
	Eastern Province	2123	49.3	40.5%	59.5%			8.5%	91.5%			3.4%	96.6%	58.777	0.000 **
	Western Province	1452	33.7	35.4%	64.6%	13.451	0.009 **	8.5%	91.5%	12.896	0.012 *	6.9%	93.1%		
	Northern Province	47	1.1	34.0%	66.0%			12.8%	87.2%			17.0%	83.0%		
	Southern Province	86	2.0	32.6%	67.4%			12.8%	87.2%			12.8%	87.2%		
Educational level	Under high school	208	4.8	36.5%	63.5%	0.855	0.836	10.1%	89.9%			11.5%	88.5%		
	High school/diploma	1255	29.2	37.8%	62.2%			9.8%	90.2%			5.2%	94.8%		
	Bachelor’s degree	2461	57.2	38.2%	61.8%			8.1%	91.9%	9.061	0.028 *	5.9%	94.1%	17.583	0.001 **
	Graduate studies	381	8.9	36.0%	64.0%			8.1%	91.9%			3.4%	96.6%		
Occupational status	Student	906	21.0	39.5%	60.5%	5.210	0.157	8.6%	91.4%	3.229	0.358	9.9%	90.1%		
	Employed (Non-health practitioner)	1416	32.9	38.9%	61.1%			8.6%	91.4%			4.1%	95.9%	40.785	0.000 **
	Employed (Health practitioner)	666	15.5	34.5%	65.5%			8.6%	91.4%			3.9%	96.1%		
	Unemployed	1317	30.6	37.1%	62.9%			8.6%	91.4%			5.5%	94.5%		

** Significant at the 0.01 level (2-tailed). * Significant at the 0.05 level (2-tailed). The percentages of Good/Poor in the rows are represented within demographic variables and read it vertically.

## Data Availability

The datasets generated and analyzed during the current study are not publicly available due to participant confidentiality but are available from the corresponding author on reasonable request.
